# Effect and mechanism of HMG-CoA reductase inhibitor on the improvement of elderly essential hypertension-induced vascular endothelial function impairment based on the JAK/STAT pathway

**DOI:** 10.1186/s13000-023-01393-x

**Published:** 2023-09-28

**Authors:** Wen Yuan, Hongjun Fan, Haibing Yang, Liang Tang, Zhiming Liu, Fan Ouyang, Wei Luo, Yong Yan

**Affiliations:** 1 Department of Neurosurgery, Central Hospital of Zhuzhou, No. 116, Changjiang South Road, Tianyuan District, 412000 Zhuzhou, Hunan China; 2 Department of Cardiology, Central Hospital of Zhuzhou, No. 116, Changjiang South Road, Tianyuan District, 412000 Zhuzhou, Hunan China

**Keywords:** 3-hydroxy-3-methylglutaryl coenzyme A (HMG-CoA) reductase inhibitor, Essential hypertension, Vascular endothelial function impairment, JAK/STAT pathway

## Abstract

**Objective:**

Our research was designed to figure out the influence and mechanism of 3-hydroxy-3-methylglutaryl coenzyme A (HMG-CoA) reductase inhibitor on the improvement of elderly essential hypertension-induced vascular endothelial function impairment based on the JAK/STAT pathway.

**Methods:**

Eighty-six elderly patients with essential hypertension were randomized into a control group (oral Amlodipine Besylate Tablets) and an observation group (oral Amlodipine Besylate Tablets + HMG-CoA reductase inhibitor atorvastatin calcium). Patients in both groups were treated with the drug for 12 weeks. Blood pressure, serum levels of inflammatory factors, and vascular endothelial function indicators, and levels of blood lipids were measured. The modeled rats were treated with atorvastatin calcium and a JAK/STAT pathway inhibitor (AG490), and the levels of cardiac function-related indices, left ventricular mass index, lipid levels, serum inflammatory factors and vascular endothelial function-related indices were detected in each group.

**Results:**

HMG-CoA reductase inhibitor improved blood pressure levels, lipid levels, serum inflammatory factor levels and cardiac function in elderly patients with essential hypertension. Both HMG-CoA reductase inhibitor and AG490 improved blood pressure levels, lipid levels, serum inflammatory factor levels and cardiac function in SHR rats. Both HMG-CoA reductase inhibitor and AG490 decreased p-JAK2/JAK2 and p-STAT3/STAT3 expression levels.

**Conclusion:**

Our study demonstrates that HMG-CoA reductase inhibitor improves elderly essential hypertension-induced vascular endothelial function impairment by blocking the JAK/STAT pathway.

**Supplementary Information:**

The online version contains supplementary material available at 10.1186/s13000-023-01393-x.

## Introduction

Despite an aging population in China, hypertension has become one of the most frequent chronic conditions in the elderly [1]. Elderly patients with hypertension usually harbor an elevated risk of damaged vascular structure or function, including decreased arterial elasticity and catecholamine responsiveness, which adversely affect blood pressure [2]. Despite notable advancement in hypertension management, the underlying pathogenic mechanisms have not been altered by the currently available treatment modalities [3]. The identification of the essential hypertension pathogenesis has progressed from a hemodynamic phenomenon associated with defective renal sodium excretion to a complicated syndrome involving metabolic, genetic, and immune system abnormalities, comprising abnormal adipose tissue distribution, endothelial dysfunction, as well as hyper-activation of the sympathetic nervous system [4]. The enormous physical and financial burden of essential hypertension on patients and society demonstrates the significance of a continued focus on seeking novel management and treatment modalities.

The 3-hydroxy-3-methylglutaryl coenzyme A (HMG-CoA) reductase inhibitors, also known as statins, were first applied for treating patients with hypercholesterolemia and hyperlipidemia [5]. Evidence has shown that in the utilization of either primary or secondary prevention, statin therapy may have relevance with a marked diminish in cardiovascular morbidity and mortality [6, 7]. Statins have a wide range of biological properties and have been indicated to reduce oxidative stress, exert anti-inflammatory effects, as well as elevate the bioavailability of nitric oxide (NO) [8]. Recently, many HMG-CoA reductase inhibitors have been discovered. Atorvastatin calcium, an HMG-CoA reductase inhibitor, is capable of reducing endogenous cholesterol synthesis, thereby resulting in a reduction in circulating (HDL-C) [9]. An article has pointed out that oral administration of atorvastatin has therapeutical effects in rats with stroke-prone spontaneously hypertensive rats through an elevated NO production due to upregulated endothelial nitric oxide synthase (eNOS) in the brain [10]. Zhang et al. have found that atorvastatin attenuates cold-induced hypertension via restraining inflammatory cytokines [11]. The JAK/STAT pathway is essential in transmitting signals from diverse cytokines together with growth factors into the nucleus, modulating gene levels, and cellular functions [12]. As reported, JAK2 and STAT3 are implicated in processes directly associated with pulmonary artery remodeling, consisting of inflammation, endothelial dysfunction, and smooth muscle proliferation [13]. Furthermore, it has been demonstrated that the JAK/STAT pathway is overactivated in the pulmonary arteries of pulmonary hypertension (PH) patients [14]. A prior paper has disclosed that atorvastatin induces cardioprotection via the JAK/STAT pathway activation in early reoxygenation [15]. Nevertheless, the interaction of atorvastatin calcium with the JAK/STAT pathway remains to be elucidated, and in our study, we aimed to unravel the mechanism of HMG-CoA reductase inhibitor on the improvement of elderly essential hypertension based on the JAK/STAT pathway, which is the novelty of our article.

## Materials and methods

### Ethical approval

The study of both clinical and animal experiments was ratified by the ethics committee of the Central Hospital of Zhuzhou (approval number: 20,181,024 and 20,190,428). The patients and their families gave their written informed consent. The disposal of animals throughout the experimental process was in accordance with ethical principles for the use of laboratory animals.

### Participants

Eighty-six elderly patients with essential hypertension admitted to the Central Hospital of Zhuzhou from March 2019 to July 2020 were included as study subjects. The general conditions of the patients are displayed in Table 1. Inclusion criteria: (1) patients aged ≥ 60 years; (2) patients were in hypertension grade I and II; (3) sustained blood pressure or 3 non-same-day blood pressure measurements of systolic blood pressure (SBP) ≥ 140 mmHg (1 mmHg = 0.133 kPa) and/or diastolic blood pressure (DBP) ≥ 90 mmHg; (4) patients had normal cardiac, pulmonary, and renal function. Exclusion criteria: (1) patients with secondary or acute hypertension; (2) patients combined with diabetes, cardiovascular disease, liver and kidney failure, and other serious diseases; (3) patients with cognitive impairment or physical dysfunction.

**Table 1:** Comparison of general data in patients

General data	Control group (n = 43)	Observation group (n = 43)	*P* value
Age (years)	69.28 ± 7.74	68.41 ± 6.49	0.598
Gender (male/female)	29/14	27/16	0.821
BMI (kg/m^2^)	25.34 ± 2.79	24.92 ± 3.88	0.566
Hypertension classification (I grade/II grade)	13/30	17/26	0.498
Mean duration of disease (year)	6.41 ± 1.13	6.25 ± 2.14	0.531
Smoking history (yes/no)	28/15	24/19	0.509
Drinking history (yes/no)	34/9	30/13	0.459

### Experimental animals

The 16-month-old SPF-class male SHR rats (genetically modified mating spontaneously hypertensive rats recognized worldwide for their genetically stable hypertension, irritability, and startle-prone nature) were utilized for establishing rat models of elderly essential hypertension, and the Wistar-Kyoto (WKY) rats of the same age with the same background was selected as normal controls. These rats (available from Vital River Laboratory Animal Technology Co., Ltd., Beijing, China) were housed in 1/cage at constant temperature (22 °C) and humidity (40-60%) with 12-h alternating light and dark. The rats were fed with conventional commercial rat chow (Laboratory Rodent Diet 5001, purchased from LabDiet, St. Louis, MO, USA) and drinking water.

### Grouping and treatment

Clinical trial: Elderly patients with essential hypertension were randomized into a control group (43 patients) and an observation group (43 patients) using the random number table method. All patients were treated with strict elution [discontinuation of previous oral antihypertensive drugs, at least 2 weeks for all types of drugs except diuretics for at least 4 weeks, and 2 weeks of placebo (a substance without pharmacological activity, provided by Novartis Pharmaceutical Co., Beijing, China) for elution treatment. Patients in the control group were given oral Amlodipine Besylate Tablets (specification: 5 mg/tablet, manufacturer: Beijing Novartis Pharmaceutical Co., Ltd.), 1 tablet/time/day; in the observation group, HMG-CoA reductase inhibitor (atorvastatin calcium; specification: 20 mg/tablet, manufacturer: Pfizer Pharmaceutical Co., Ltd.) was given on the basis of the control group, 20 mg/time, 1 time/day, taken orally at bedtime. Patients in both groups were treated with the drug for 12 weeks [16–18].

Animal experiment: After 1 week of adaptive feeding, SHR rats were allocated into the model group (SHR rats without any treatment), HMG-CoA reductase inhibitor group (abbreviated as the HMG-CoA group, SHR rats were treated with atorvastatin calcium), AG490 group (SHR rats were given the JAK/STAT pathway inhibitors), and HMG-CoA + AG490 group (SHR rats were given atorvastatin calcium combined with the JAK/STAT pathway inhibitors) (n = 10). Ten WKY rats were selected as the normal group. Rats in the HMG-CoA and HMG-CoA + AG490 groups were given atorvastatin calcium 10 mg/kg/d in 1 mL distilled water by gavage, rats in the normal and model groups were given the same amount of distilled water by gavage, while rats in the AG490 and HMG-CoA + AG490 groups were given AG490 (5 mg/kg/d in 1 mL saline) by gavage daily for 12 weeks [19–21].

### Blood pressure testing

In patients of both groups, SBP and DBP were measured using a blood pressure monitor (Omron, Dalian, China) before and after 12 weeks of treatment. Blood pressure measurements were implemented at 8–10 a.m. All patients took a sitting position, emptied their bladders, stopped smoking and drinking stimulating beverages within 30 min before measurement, and took the right upper arm for measurement after 20 min of meditation. Three consecutive measurements were taken for each patient, with an interval of 3 min each, and the mean value was taken.

After 12 weeks of treatment, SBP and DBP in the tail artery of each group of rats in the awake state were measured from 8:00 a.m. to 11:00 a.m. using a fully automatic rat non-invasive blood pressure measurement system (BP-300 A, Techman, Chengdu, China). Each rat was measured three times consecutively with an interval of 3 min each time, and the average value was taken. The standardized measurements were performed by dedicated personnel to minimize the error.

### Echocardiogram measurement

After 12 weeks of treatment, the rats in each group were weighed and anesthetized by intraperitoneal injection of pentobarbital sodium (40 mg/kg). The left ventricular (LV) long axis, left ventricular internal diameter at end-diastole (LVIDd), and left ventricular internal diameter at end-systolic (LVIDs) were averaged over 3 consecutive cardiac cycles using an Acuson Sequoia 512 ultrasound system (Siemens, Germany) with a probe frequency of 14 MHz and a depth of 3 cm at a speed of 100–200 mm/s. Left ventricular ejection fraction (LVEF) and left ventricular shortening fraction (LVFS) were also measured to evaluate the systolic function of the left ventricle [22].

### Measurement of left ventricular mass index (LVMI)

Immediately after all of the above measurements, the rat’s thorax was opened and the heart was quickly obtained. Upon rinsing with ice saline, the atria and right ventricular free wall were incised along the atrioventricular ring. After drying with filter paper, the remaining septum and left ventricular free wall were weighed as the LV mass. LVMI = LV mass/body weight (mg/g) [23].

### Evaluation of inflammatory factors

Fasting venous blood was harvested before and after treatment from patients in the control and observation groups and tail vein blood from mice in each group, with or without EDTA anticoagulation, centrifuged at 3000 r/min for 10 min, then serum or plasma was separated and stored at -20℃. Serum interleukin-6 (IL-6), tumor necrosis factor-α (TNF-α) and hypersensitive-C-reactive protein (hs-CRP) levels were estimated by ELISA kits (Nanjing Jiancheng Institute of Biological Engineering, Nanjing, China) [24, 25].

### Blood lipid detection

Serum total cholesterol (TC), triglyceride (TG), LDL-C, and HDL-C levels were assessed before and after 12 weeks of treatment in both groups of patients and in each group of rats using a fully automated biochemical analyzer (Senlo, Zhuhai, China) [26].

### Vascular endothelial function index test

Plasma endothelin (ET), NO, and eNOS levels were measured by ELISA kits (Nanjing Jiancheng Institute of Biological Engineering) before and after treatment in two groups of patients and each group of mice [27].

### Western blot

Total protein was extracted from rat blood samples using RIPA protein lysate (Solarbio, Beijing, China). Protein content was quantified using the BCA Protein Concentration Assay Kit (Solarbio). The proteins were separated by 8% SDS-PAGE and transferred to PVDF membranes, which were closed with 5% skimmed milk at ambient temperature for 2 h. Next, the membranes were subjected to cultivation with primary antibodies against JAK2 (1:500), p-JAK2 (1:1000), STAT3 (1:500), p-STAT3 (1:1000) and β-actin (1:1000) (all from Cell Signaling Technology, Inc., USA) overnight at 4 °C, followed by 1 h-cultivation with horseradish peroxidase-labeled goat anti-rabbit IgG secondary antibody at 37 °C. Protein bands were tested using an ultra-sensitive enhanced chemiluminescence kit (Beyotime, Shanghai, China). The grayscale values of each protein band were analyzed using Image-Pro Plus (version 6.0, Media Cybernetics, Inc.).

### Statistical analysis

All data were statistically processed with SPSS 21.0 (IBM SPSS Statistics, Chicago, IL, USA). Measurement data were denoted as mean ± standard deviation, with the t-test for two-group comparisons and one-way ANOVA for multi-group comparisons. Enumeration data were depicted as percentages or rates, and group comparisons were made using Fisher’s exact test or χ^2^ test. A notable difference was found with a *p*-value below 0.05.

## Results

### General data for patients

The general data of age, gender, body mass index, hypertension classification, mean duration of disease, smoking history, and drinking history exhibited no marked differences between the control and observation groups (all *p* > 0.05, Table 1).

### Blood pressure and blood lipid levels in patients before and after treatment

Before treatment, no distinct differences were noted in blood pressure levels (SBP and DBP) and lipid-related indices (TC, TG, LDL-C, and HDL-C) in patients between the control and observation groups (all *p* > 0.05). In both groups, SBP, DBP, TC, TG, and LDL-C showed lower levels while HDL-C exhibited high levels after treatment in comparison to those before treatment (all *p* < 0.05). After treatment, SBP, DBP, TC, TG, and LDL-C were significantly lower while HDL-C was significantly higher in the observation group versus those in the control group (all *p* < 0.05) (Table 2).

**Table 2:** Comparison of blood pressure and blood lipid levels before and after treatment between the two groups ($\stackrel{-}{x}$ ± SD)

Indicator		Control group (n = 43)	Observation group (n = 43)
SBP (mmHg)	Before treatment	156.23 ± 7.61	155.40 ± 6.87
	After treatment	141.74 ± 6.62*	130.17 ± 5.69*#
DBP (mmHg)	Before treatment	98.18 ± 6.35	96.76 ± 6.30
	After treatment	90.65 ± 5.23*	75.36 ± 5.52*#
TC (mmol/L)	Before treatment	6.67 ± 0.91	6.72 ± 0.85
	After treatment	5.14 ± 0.68*	4.06 ± 0.65*#
TG (mmol/L)	Before treatment	2.39 ± 0.34	2.45 ± 0.28
	After treatment	1.88 ± 0.25*	1.42 ± 0.19*#
LDL-C (mmol/L)	Before treatment	3.84 ± 0.65	3.90 ± 0.81
	After treatment	3.06 ± 0.72*	2.58 ± 0.55*#
HDL-C (mmol/L)	Before treatment	1.03 ± 0.41	1.02 ± 0.34
	After treatment	1.24 ± 0.25*	1.42 ± 0.31*#

Note: * *p* < 0.05 vs. Before treatment; # *p* < 0.05 vs. Control group

### Serum inflammatory factor levels in patients before and after treatment

There was no marked difference in the levels of serum inflammatory factors (IL-6, TNF-α, and hs-CRP) in the control and observation groups before treatment (all *p* > 0.05). Decreased levels of IL-6, TNF-α, and hs-CRP were found in both groups after treatment compared with those before treatment (all *p* < 0.05). After treatment, reduced levels of IL-6, TNF-α, and hs-CRP were noted in the observation group versus those in the control group (all *p* < 0.05) (Table 3).

**Table 3:** Comparison of serum levels of inflammatory factors before and after treatment between the two groups ($\stackrel{-}{x}$ ± SD)

Indicator		Control group (n = 43)	Observation group (n = 43)
IL-6 (ng/L)	Before treatment	15.15 ± 3.27	15.40 ± 3.65
	After treatment	9.38 ± 2.03*	7.54 ± 1.12*#
TNF-α (ng/L)	Before treatment	19.35 ± 3.69	19.57 ± 3.51
	After treatment	15.41 ± 2.15*	13.22 ± 2.08*#
hs-CRP (mg/L)	Before treatment	8.87 ± 1.86	8.92 ± 2.18
	After treatment	6.28 ± 1.50*	3.08 ± 1.02*#

Note: * *p* < 0.05 vs. Before treatment; # *p* < 0.05 vs. Control group

### Vascular endothelial function indices in patients before and after treatment

Before treatment, the levels of vascular endothelial function indices (ET, NO, and eNOS) exhibited no notable difference in patients between the control and observation groups (all *p* > 0.05). After 12 weeks of treatment, the levels of NO and eNOS in both groups were higher whereas the levels of ET were lower than those before treatment (all *p* < 0.05). After treatment, decreased levels of ET, along with elevated levels of NO and eNOS, were observed in the observation group relative to those in the control group (all *p* < 0.05) (Table 4).

**Table 4:** Comparison of indices of vascular endothelial function before and after treatment between the two groups ($\stackrel{-}{x}$ ± SD)

Indicator		Control group (n = 43)	Observation group (n = 43)
ET (ng/L)	Before treatment	87.23 ± 9.32	88.36 ± 8.70
	After treatment	67.14 ± 7.95*	58.24 ± 7.16*#
NO (mmol/L)	Before treatment	43.18 ± 5.24	43.27 ± 5.42
	After treatment	54.08 ± 6.34*	66.25 ± 6.68*#
eNOS (U/L)	Before treatment	45.50 ± 7.88	47.36 ± 8.15
	After treatment	50.31 ± 9.40*	65.64 ± 10.46*#

Note: * *p* < 0.05 vs. Before treatment; # *p* < 0.05 vs. Control group

### HMG-CoA reductase inhibitor mediates the JAK/STAT3 pathway to improve vascular endothelial function impairment in elderly patients with essential hypertension

From the aforesaid clinical trials and related articles [28, 29], it is clear that HMG-CoA inhibitors are a group of drugs widely applied in hypertension, hyperlipidemia, and coronary artery disease, which can improve endothelial functional impairment. Also, evidence has shown that the JAK/STAT pathway, which is activated during the progression of essential hypertension, exacerbates the development of vascular inflammation [30]. Therefore, we propose the hypothesis that HMG-CoA reductase inhibitors may improve endothelial dysfunction of essential hypertension in the elderly by blocking the JAK/STAT g pathway.

**Fig. 1:**
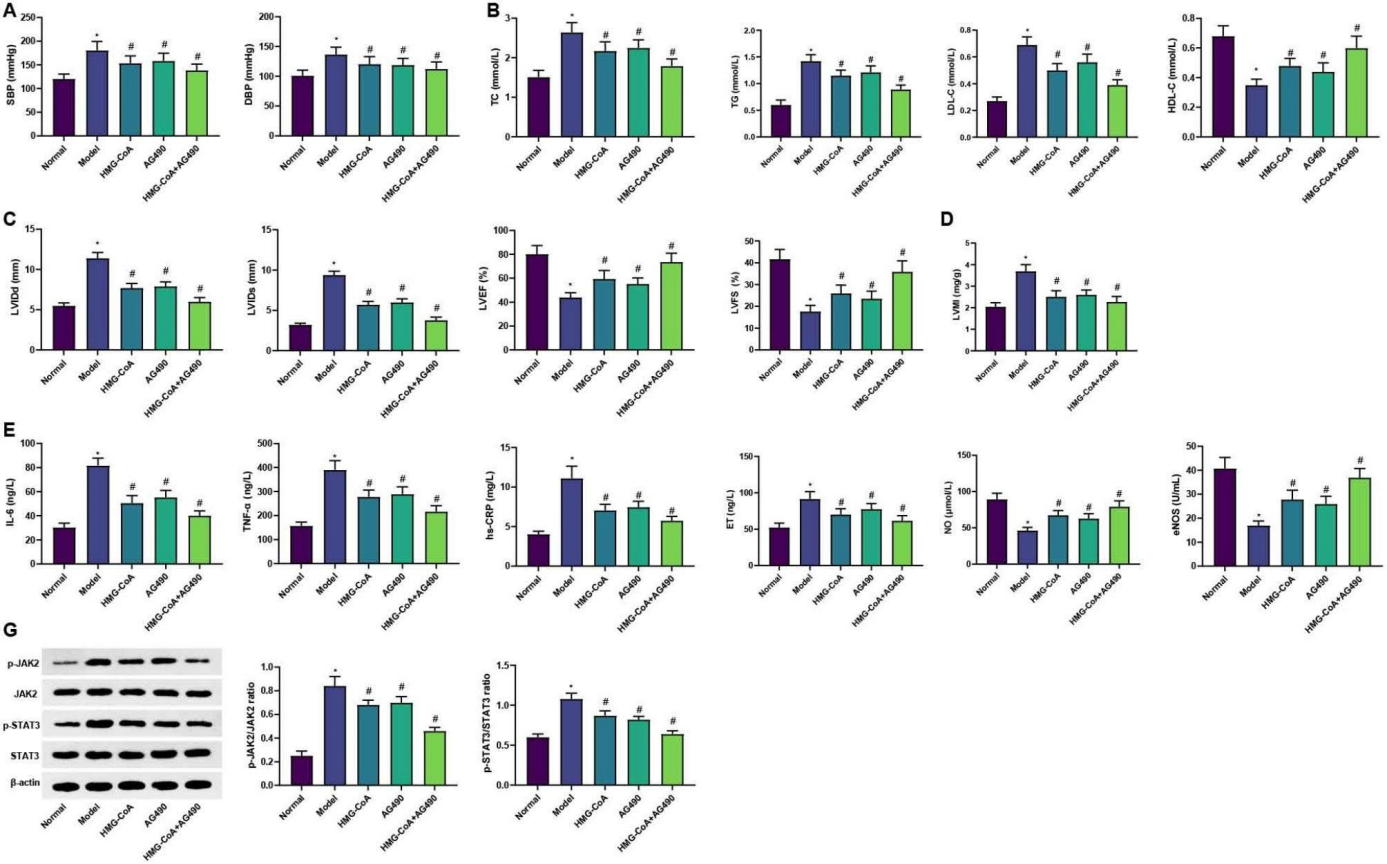
HMG-CoA reductase inhibitor mediates the JAK/STAT3 pathway to improve vascular endothelial function impairment in elderly patients with essential hypertension. **A**. Blood pressure (SBP and DBP) levels in each group of rats. **B**. Blood lipid levels in each group of rats were detected by an automatic biochemical analyzer. **C**. Ultrasound detection of cardiac function-related indices in each group of rats. **D**. Left ventricular mass index in each group of rats. **E**. ELISA detection of inflammatory factor levels in each group of rats. **F**. ELISA detection of vascular endothelial function-related indices in each group of rats. **G**. Western blot detection of p-JAK2, JAK2, p-STAT3, and STAT3 protein expression in each group of rats. n = 10. * *p* < 0.05 vs. Before treatment; # *p* < 0.05 vs. Control group

To test this hypothesis, we treated SHR rats with an HMG-CoA reductase inhibitor (atorvastatin calcium) and a JAK/STAT pathway inhibitor (AG490). The blood pressure levels (SBP and DBP), lipid levels (TC, TG, LDL-C, and HDL-C), cardiac function-related indices (LVIDd, LVIDs, LVEF, and LVFS), LVMI, serum inflammatory factor levels (IL-6, TNF-α, and hs-CRP), and vascular endothelial function indices levels (ET, NO, and eNOS) were evaluated. Compared with the normal group, there exhibited elevated levels of SBP, DBP, TC, TG, LDL-C, LVIDd, LVIDs, LVMI, IL-6, TNF-α, hs-CRP, and ET, and reduced levels of HDL-C, LVEF, LVFS, NO, and eNOS in the model group (all *p* < 0.05). Reduced levels of SBP, DBP, TC, TG, LDL-C, LVIDd, LVIDs, LVMI, IL-6, TNF-α, hs-CRP, and ET, and elevated levels of HDL-C, LVEF, LVFS, NO, and eNOS were noted in the HMG-CoA, AG490, and HMG-CoA + AG490 groups versus those in the model group (all *p* < 0.05). Among them, rats in the HMG-CoA + AG490 group showed the best improvement in the above indices (Fig. 1A-F). The echocardiography results of each group are shown in Supplementary Fig. 1.

Furthermore, the protein expression levels of JAK/STAT pathway-related factors in each group were tested by western blot (Fig. 1G). Higher p-JAK2/JAK2 and p-STAT3/STAT3 protein levels were observed in the model group relative to the normal group (both *p* < 0.05). In contrast to the model group, p-JAK2/JAK2 and p-STAT3/STAT3 protein levels were reduced in the HMG-CoA, AG490, and HMG-CoA + AG490 groups, among which the levels in the HMG-CoA + AG490 group had the greatest decrease. The above studies suggest that HMG-CoA reductase inhibitors improve the development of essential hypertension in the elderly by blocking the JAK/STAT pathway.

## Discussion

Essential hypertension is featured with persistently elevated blood pressure levels, which in turn results in target organ damage together with an enhanced risk of cardiovascular disease [31]. HMG-CoA reductase inhibitors (statins) are able to improve endothelial function and play antiproliferative roles on systemic vessels’ vascular smooth muscle cells [32]. Specifically, statins improve endothelial function via elevating endothelial NO production and post-transcriptional eNOS, reducing cholesterol and ET-1, as well as diminishing the affinity for angiotensin-I receptors [33]. To further validate the therapeutic influence of HMG-CoA reductase inhibitor on hypertension, we conducted this work and found that HMG-CoA reductase inhibitor improved elderly essential hypertension-induced vascular endothelial function impairment by blocking the JAK/STAT pathway.

The activation of inflammatory pathways is a crucial pathological event in hypertension development [34]. Lipids have been demonstrated to impact an inflammatory reaction, thereby resulting in the expansion of metabolic syndrome, which serves as a risk parameter in cardiovascular disease pathogenesis, including hypertension [35]. Levels of inflammatory mediators (IL-6, TNF-α, and hs-CRP) in pregnancy-induced hypertension (PIH) patients have a positive link with the SBP and a poor fetal prognosis of patients, which could be utilized as a parameter of prognosis estimation of PIH patients [25]. Also, a recent article has disclosed higher levels of IL-6, TNF-α, and hs-CRP in uncomplicated hypertension [36]. TC, TG, and LDL-C serum levels are increased whereas HDL-C serum levels are decreased with the progression of hypertensive disorder complicating pregnancy (HDP) [37]. Another study has unveiled that elevated TC, LDL-c, and non-HDL-c are related to hypertension incidence in Chinese adult males [38]. All the aforesaid references support that inflammatory mediators and levels of blood lipids are critical factors for assessing essential hypertension.

In our work, we found that HMG-CoA reductase inhibitor improved blood pressure levels, lipid levels, serum inflammatory factor levels and cardiac function in elderly patients with essential hypertension. Many previous studies coincide with the results of our article. For blood pressure, statin treatment in combination with antihypertensive treatment decreases blood pressure in contrast to antihypertensive treatment combined with a hypocholesterolemic diet [39]. For the indices of vascular endothelial function, statins impact NO production in the brain via mitigating iNOS and advancing eNOS activity [40]. Besides, atorvastatin alleviates arsenic-stimulated hypertension via improving aortic NO signaling and lipid profile, and restoring vascular redox homeostasis [41]. In the meantime, statins also reduce the ET-1 mRNA levels and ET-1 synthesis in vitro [42]. For lipid levels, it has been demonstrated that atorvastatin reduces total-C, TG, LDL-C, VLDL-C, non-HDL-C, and IDL-C, and generates variable increases in apolipoprotein A-1 and HDL-C [43]. For inflammatory factor levels, in a clinical study, HMG-CoA reductase inhibitors are beneficial for the outcome of epilepsy, and statin reduces the risk of hospitalization due to their anti-inflammatory properties [44]. Moreover, atorvastatin has a beneficial impact on endothelial function and inflammation, which is reflected by decreased serum levels of ICAM-1 and TNF-α levels in hypertension [45]. All of these studies highlighted the therapeutic role of HMG-CoA reductase inhibitors, especially atorvastatin, in hypertension.

Also, our research signified that both HMG-CoA reductase inhibitor and AG490 improved blood pressure levels, lipid levels, serum inflammatory factor levels and cardiac function in SHR rats. Besides, both HMG-CoA reductase inhibitor and AG490 decreased p-JAK2/JAK2 and p-STAT3/STAT3 expression levels. In a bleomycin-induced PH animal model, p-JAK2/p-STAT3 is found to be upregulated and localized in pulmonary arteries [46]. Both p-JAK and p-STAT activate diverse proteins and transcription factors, all of which are involved in modulating proliferation and apoptosis resistance, which contributes to the development of PH [47]. A previous paper has unraveled that the activation of the JAK/STAT pathway stimulates NOS activity, consistent with the elevated basal JAK/STAT and NOS activities in SHR myocytes [48]. Similar to our paper, Koike A et al. supported that statins suppress the levels of IFN-β and IFN-stimulated genes in hyperlipidemic mice via the blockage of the JAK/STAT pathway [49]. Another study has elucidated that HMG-CoA reductase inhibitors are promising medications for treating cardiac hypertrophy via the JAK/STAT pathway [50].

In summary, our study underscores that HMG-CoA reductase inhibitor improves elderly essential hypertension-induced vascular endothelial function impairment by blocking the JAK/STAT pathway. HMG-CoA reductase inhibitor, especially atorvastatin, could act as a suitable therapy for essential hypertension in elderly patients, and and JAK/STAT inhibition might be promising in patients with hypertension. Nevertheless, further experiments are necessary for the validation of our findings.

### Electronic supplementary material

Below is the link to the electronic supplementary material.

Supplementary Material 1

## Data Availability

The data that support the findings of this study are available from the corresponding author upon reasonable request.
