# Supplementary figures and images for: Effect and mechanism of HMG-CoA reductase inhibitor on the improvement of elderly essential hypertension-induced vascular endothelial function impairment based on the JAK/STAT pathway
Source: Diagn Pathol. 2023 Sep 28;18:108. doi: 10.1186/s13000-023-01393-x (PMC10536732; doi:10.1186/s13000-023-01393-x)

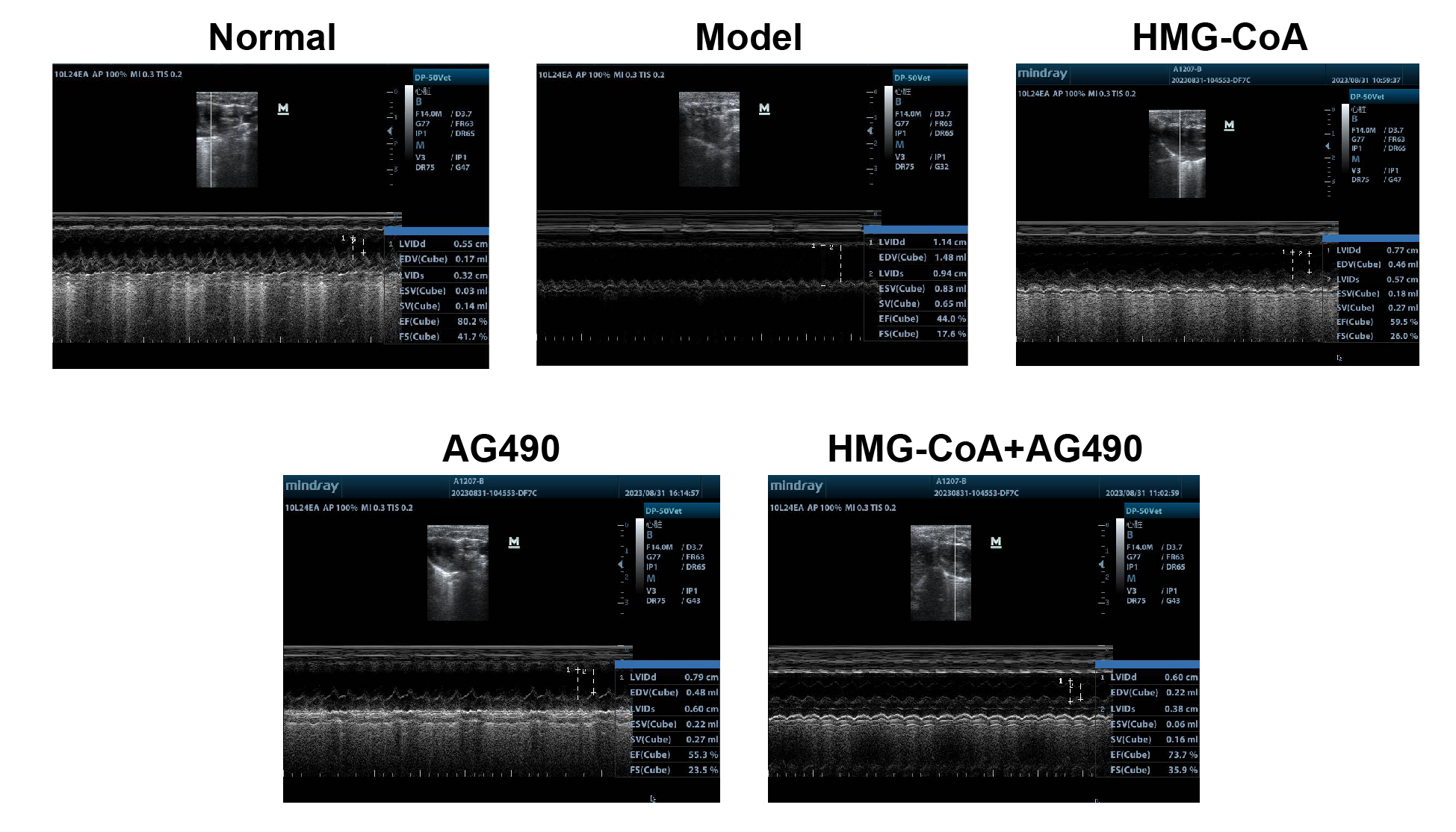

Supplement: Supplementary file 1 — Supplementary Material 1 [file 13000_2023_1393_MOESM1_ESM.jpg]
